# BMI trajectory in adulthood in relation to all-cause and cause-specific mortality: A retrospective cohort study in Taiwan

**DOI:** 10.1371/journal.pone.0295919

**Published:** 2023-12-20

**Authors:** Po-Wei Chiu, Tsung Yu, Shikha Kukreti, Carol Strong

**Affiliations:** Department of Public Health, National Cheng Kung University Hospital, College of Medicine, National Cheng Kung University, Tainan, Taiwan; University of Montenegro, MONTENEGRO

## Abstract

A dynamic change of weight over time has been known as an important factor that impacts mortality risk. The aims of this study were to identify the heterogeneity of BMI trajectory groups and to examine the association of the trajectories of BMI and all-cause and cause-specific mortality. The data for this study were obtained from a large prospective cohort study in Taiwan between 1998 and 2019 that was linked to the National Death Registry for death information. The participants were stratified into four groups by age and gender; self-reported demographics and measured BMI data were used. We used group-based trajectory analysis to identify the distinct trajectories of changes in BMI. A Cox proportional hazards model was used to assess the hazard ratio (HR) of all-cause and cause-specific mortality risk. Data were analyzed in April 2020 and included 89,886 participants. Four trajectory groups were identified by the pattern of BMI change over time. Our study shows that different trajectories were associated with mortality. Our findings suggest that the mortality risk differs in each trajectory group and in each age and gender stratification. It appears that obesity is a protective factor in cancer-related mortality in females but not in males in group of old age participants; low-normal weight is a risk factor in respiratory-related mortality in all participants. Our findings can be used to suggest the appropriate BMI in each age and gender groups and thereby earlier health interventions can be taken to avoid mortality.

## Introduction

A dynamic change of weight over time has been known as an important factor of mortality risk, especially among older adults [1]. Body mass index (BMI) trajectory studies can highlight the importance of initial weight status and the amount of weight gain in relation to mortality. In a study that examined BMI trajectory in an elderly adult sample in the United States (US), small weight gain (defined as less than 10 kilograms) was not associated with increasing mortality risk, regardless of the patients’ initial BMI, whereas a large weight gain increased the mortality risk when the initial BMI was greater than 35 kilograms [2]. However, most of the trajectory studies focused on all-cause mortality. It is important to investigate the different causes of death among groups of different BMI because cancer and cardiovascular disease deaths may be related to BMI [3,4].

The association between change of BMI and mortality risk has been reported as “reverse J shape” since underweight and obesity are associated with increased mortality risk [5]. Overweight, however, has sometimes been found to have a protective effect to all-cause mortality rate among elderly, which is known as the “obesity paradox” [6–8]. Overweight groups have the lowest risk of death in both Japan and Western populations [5]. In several US samples, the association differs by the amount of weight change. Modest weight gains are associated with a significantly decreased mortality risk, whereas excessive weight gains predict a significantly increased mortality risk [5]. The literature regarding the obesity paradox is not consistent. Some studies have evidence against this phenomenon [9], and consider it as a phenomenon of reverse causality that a low BMI population may undergo illness such as cancer or severe infection.

Several types of diseases are negatively related to obesity and overweight such as cardiovascular disease (CVD) [10,11], certain types of cancer such as breast, colon, and uterine cancers [12]. Yet, obesity and overweight are protective factors for respiratory diseases, such as chronic obstructive pulmonary disease (COPD) and asthma [13]. Further, differences in the BMI level, pattern of weight change, and cause of death have been found in different age and gender groups using group-based trajectory analysis [14], a method that identifies the distinct trajectories of changes in BMI. Overweight or obese trajectory groups in younger adulthood (age 40–60) were more likely to have numerous health conditions especially on cardiovascular disease and to have higher mortality rates. Overweight or obesity in older age (age 60+) groups, however, were found to be protective factors against mortality [15]. In addition, one study revealed that men tended to have a higher risk of developing life-threatening conditions—especially obesity-related deaths such as heart disease and diabetes. Conversely, women were more susceptible to debilitating but non-fatal conditions such as arthritis and depression [16].

In order to better access the effects of weight pattern changes over time, it is essential to examine the all-cause and cause-specific mortality risk consequences of BMI trajectories. Identification of cause-specific mortality risk in different BMI trajectories can facilitate our understanding of the details behind the “obesity paradox.”

The present study has two specific aims. First, we applied group-based trajectory models to explore the heterogeneity of the BMI trajectory throughout eighteen years in adulthood among Taiwanese adults over 40 years of age. Studies have identified different trajectory groupings and mortality rates for men and women and for adults in ages 40–60 compared to age 60+ [15]. For example, in a sample of Canadian adults, men tended to maintain a stable BMI trajectory; women were more likely to experience a decrease in BMI over time, which may be related to the differences in hormones and body composition between genders [15]. Therefore, we hypothesized that different trajectory groups would be identified by age and sex groups. Second, we examined the relationship between the trajectories of BMI and all-cause and cause-specific mortality. Specifically, some causes of deaths such as CVD and cancer are associated with the trajectory that is consistently overweight or obese, but respiratory disease as cause of death is associated with a lower BMI trajectory. We also explored the differences in the association between BMI trajectory and mortality stratified by gender and age groups.

## Materials/Subjects and methods

### Participants

This research was conducted using the Taiwan Mei-Jao (MJ) Cohort resource, which is a longitudinal, population-based health dataset run by the MJ Health Management Institution, Taiwan [17]. The details of the MJ Cohort population and data collection are reported elsewhere [18–21]. According to the cohort profile [22], MJ is a membership which is a private fee-for-service company offering comprehensive health screening programs. The MJ cohort has enrolled around 600,000 Taiwanese individuals since 1994. Participants of the MJ cohort receive health examinations including self-reported questionnaires on medical, social, and family history as well as demographic information, and undergo a series of medical tests that include complete blood cell count, differential count, biochemistry tests, and physical examinations. The MJ Health Management Institution was authorized by every participant to process the data produced from their medical screening through the signing of a consent form [22].

**Fig 1:**
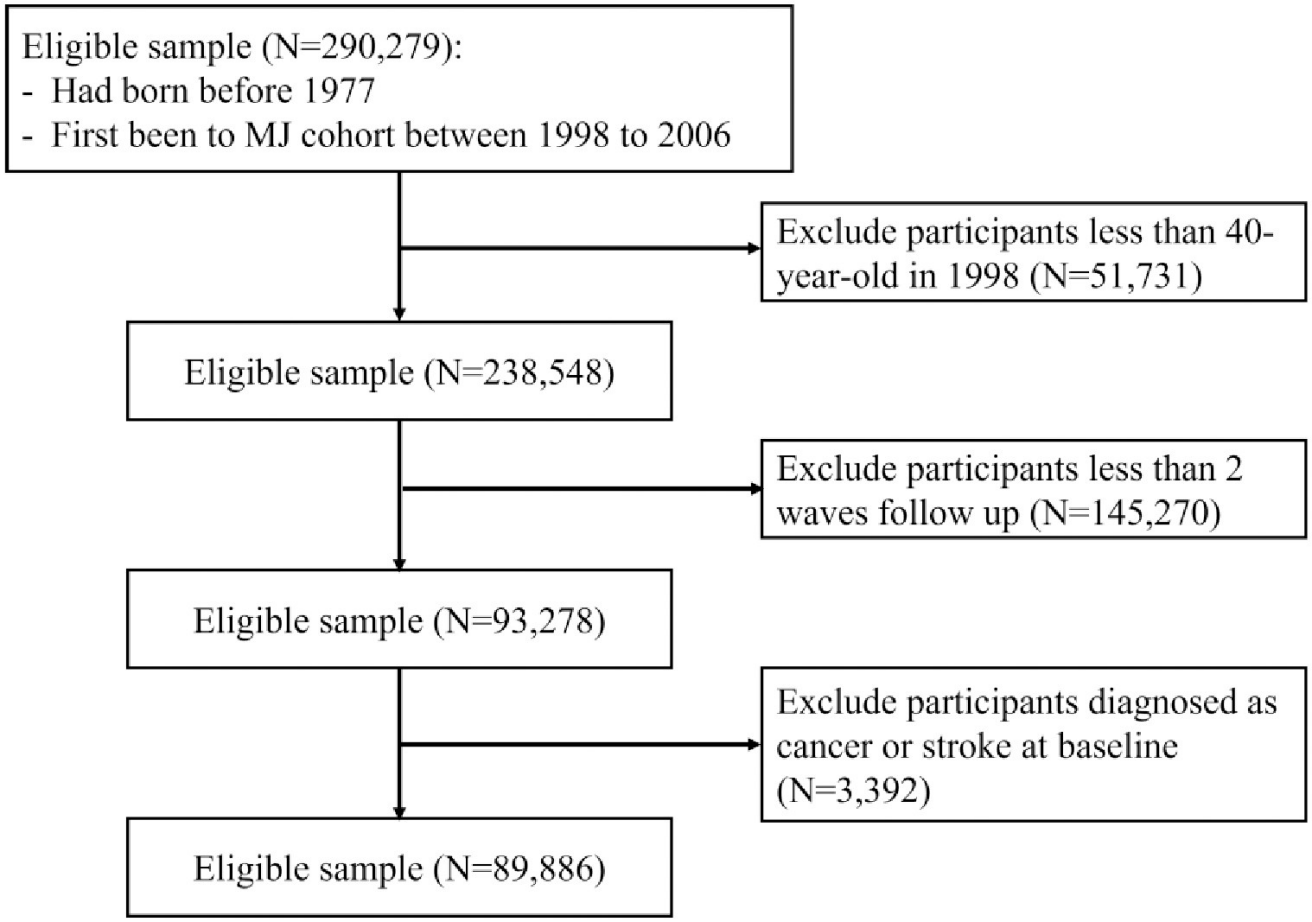
Patient attrition and cohort selection. Inclusion and exclusion criteria show cohort selection for the MJ cohort dataset.

The details of participant selection for the present study are shown in Fig 1. The database accumulated 290,279 participants who first went to the MJ cohort between 1998 and 2006. Of the included participants, we obtained all their data collected from the year they first went to the MJ cohort to year 2017. Setting three years as an interval, we built a 7-wave longitudinal dataset; an interval of a seventh wave has only 2 years, 2016–2017). If two or more measurements for one person were accessible within one interval, the measurement closest to the center of the interval was chosen. We excluded those who were less than 40 years old in 1998 (n = 51,731), less than two waves follow-up (n = 145,270), and who had ever been diagnosed at baseline with any of the following self-reported conditions: cancer, stroke (n = 3,392). The final study sample included 89,886 participants. We separated the participants into 4 groups by sex (male and female) and age (40- to 60-year-old and more than 60-year-old).

Death information was based on the National Death Registry obtained from the Ministry of Health and Welfare, Taiwan, and was linked to the MJ cohort. All deaths were identified from death certificates and were confirmed by trained physicians. Follow-up time for mortality started at the date of each participant’s first measurement and was censored by October 31, 2019, the date when we linked death information to the MJ cohort. We categorized the cause of mortality into three leading causes: “cancer,” including all kinds of cancer, “cardiovascular disease,” and “respiratory disease.” All other causes of death were grouped into the category “Other.”

### Mortality

This analysis focused on 2 separate outcome measures: (1) hazard of all-cause mortality in distinct groups, and (2) hazard of cause-specific mortality in distinct groups.

### BMI

Body height and weight were measured at every follow-up visit for each participant and BMI was calculated by dividing the weight (in kilograms) by their height squared (in meters). According to the BMI classification by the World Health Organization (WHO), BMI above 30 (kg/m^2^) should be classified as obesity, whereas BMI between 25 and 30 should be classified as overweight; BMI below 18.5 was classified as underweight [23].

### Covariate variables

Age, smoking status, alcohol consumption, educational level, and physical activity at baseline were obtained from the self-reported questionnaire from the MJ cohort and were incorporated as covariates in the present study. Age was defined at the year of 1998. Smoking status and alcohol consumption were both classified into three categories: never smoker/drinker, former smoker/drinker, and current smoker/drinker. The educational level was classified into two categories: high school or less, and college or above. Physical activity was classified into three categories: seldom (exercise less than two hours a week), sometimes (exercise between two and five hours a week), and frequent (exercise more than five hours a week).

### Statistical methods

Descriptive statistics (mean, standard deviation, and percentages) were used to summarize the demographic and clinical characteristics of participants. We used a group-based trajectory model with maximum likelihood estimation to identify the distinct trajectories of changes in BMI using the SAS PROC TRAJ program (SAS Institute, Inc., Cary, North Carolina) [24]. As recommended, we estimated models with 2–5 trajectories by assuming linear, quadratic, and cubic patterns of change in BMI over time and the best-fitting model (the number of distinct trajectories and the patterns of change in BMI) was determined on the basis of Bayesian Information Criterion scores(BIC) [25].

The hazard ratio (HR) of all-cause mortality was analyzed using a Cox proportional hazards model, including BMI trajectory groups, and the previously mentioned covariates including age, smoking status, alcohol consumption, educational levels, and physical activity. We used the time from baseline as the time scale to parameterize the baseline hazard function [26] because different birth cohorts were observed at different ages. The analyses were performed using the SAS PROC PHREG program.

A cause-specific hazards model was used to assess the HR of cause-specific mortality. This model can be estimated by censoring participants with the competing event and then fitting the standard Cox proportional hazards model [27]. The analyses were performed using the SAS PROC PHREG program.

## Results

Of all 89,886 participants, 53.3% were women, 82.5% were between 40 and 60 years of age (Table 1). Mean age was 55.2 (year-old) and mean BMI was 24.1 (kg/m^2^). The mean follow-up time was 16.8 years; 14.2 years for participants aged over 60 and 17.3 years for participants aged between 40 and 60. A quarter of participants had a college degree, but a much lower proportion of women aged over 60 obtained a college degree (3.8%). In terms of lifestyle factors, women were more likely to be never smokers or never drinkers, regardless of age groups. Overall, 38.3% had a seldom level of physical activity, 18.6% were sometimes, and 35.5% were frequent.

**Table 1:** Baseline characteristics by the subgroups classify by gender and age.

	Male aged over 60	Male aged 40–60	Female aged over 60	Female aged 40–60	Total
People count, number	8140	33790	7621	40335	89886
Age, mean(SD), years	70.1 (5.3)	51.9 (6.5)	69.5 (5.3)	52.3 (6.2)	55.2 (9.1)
Person-year, mean(SD), person-years	14.2 (5.4)	17.2 (3.6)	15.5 (4.9)	17.4 (3.3)	16.9 (3.9)
Number of people died during follow-up	4498 (55.3)	3987 (11.8)	3100 (40.7)	2863 (7.1)	14448
Cause of death					
Cancer(%)^a^	1366 (30.4)	1805 (45.3)	813 (26.2)	1437 (50.2)	5421 (37.5)
Cardiovascular disease(%)^a^	1062 (23.6)	748 (18.8)	801 (25.8)	438 (15.3)	3049 (21.1)
Respiratory disease(%)^a^	652 (14.5)	197 (4.9)	286 (9.2)	110 (3.8)	1245 (8.6)
Others(%)^a^	1418 (31.5)	1237 (31)	1200 (38.7)	878 (30.7)	4733 (32.7)
BMI, mean(SD)	23.7 (3.1)	24.4 (3.0)	24.6 (3.5)	23.7 (3.4)	24.0 (3.3)
Smoking status, number(%)^b^					
Never	3504 (45.6)	15611 (48.7)	6893 (95.5)	36416 (95.3)	62424 (73.3)
Former	1648 (21.4)	4114 (12.8)	111 (1.5)	312 (0.8)	6185 (7.3)
Current	2535 (33.0)	12351 (38.5)	215 (3.0)	1475 (3.9)	16576 (19.5)
Alcohol comsuption, number(%)^c^					
Never	4546 (61.3)	18517 (59.1)	6459 (95.8)	32556 (91.9)	62078 (76.7)
Former	886 (12.0)	2061 (6.6)	97 (1.4)	629 (1.8)	3673 (4.5)
Current	1981 (26.7)	10779 (34.4)	189 (2.8)	2254 (6.4)	15203 (18.8)
Education level, number(%)^d^					
High school or less	6046 (79.0)	18720 (58.1)	6950 (96.0)	31500 (81.6)	63216 (73.8)
College or above	1604 (21.0)	13487 (41.9)	288 (4.0)	7088 (18.4)	22467 (26.2)
Physical activity, number(%)^e^					
Seldom	2372 (31.9)	12370 (39.7)	2612 (37.0)	17065 (45.8)	34219 (41.4)
Sometimes	1113 (15.0)	6798 (21.8)	1201 (17.0)	7631 (20.5)	16743 (20.3)
Frequent	3952 (53.1)	11983 (38.5)	3243 (46.0)	12526 (33.7)	31704 (38.4)

^a^The denominator of the proportion is the total number of death for each column.

^b^missing data 4701 (5.0%).

^c^missing data 8932 (9.9%).

^d^missing data 4203 (4.6%).

^e^missing data 7020 (7.8%).

As shown in Table 1, 14,448 participants (16.1%) died during follow-up; the majority of deaths were caused by cancer (37.5%), followed by others (32.7%), cardiovascular disease (21.1%), and respiratory disease (8.6%). Both men and women in the age group of 40–60 had a higher proportion of cancer deaths than their counterparts in the age group of 60 or above (men: 45.3% (aged 40–60) vs. 30.4% (aged over 60); women: 50.2% (aged 40–60) vs. 26.2% [aged over 60]).

**Fig 2:**
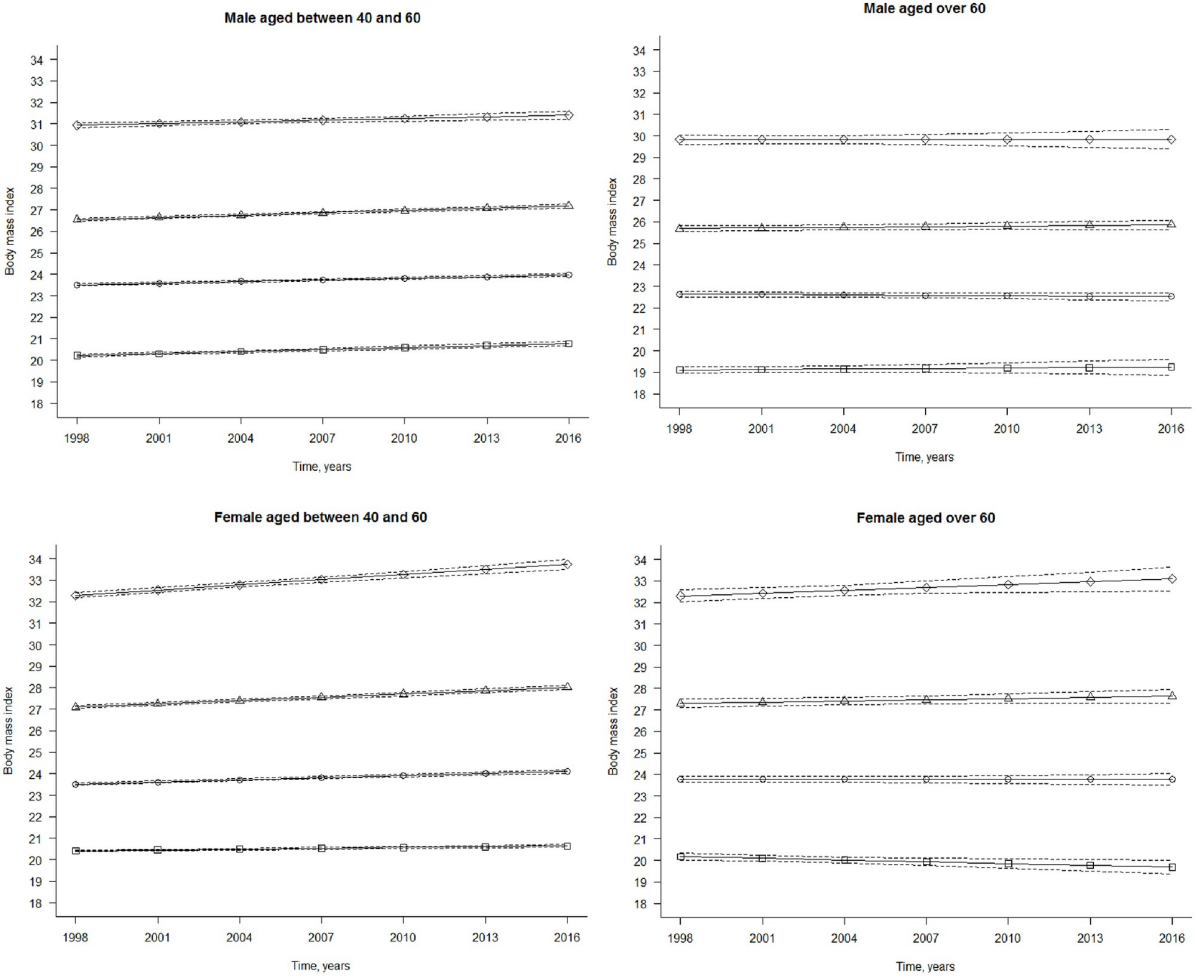
Body mass Índex trajectòries for the 4-group model over 19 years, adults from MJ cohort, 1998–2017. Solid Unes indícate the mean valúes of body mass índex (weight (kg)/height (m)^2^) for members in the groups; dashed lines, 95%confidence intervals, (a) Male aged between 40 and 60. The trajectòries are as follows (from top to bottom): diamond, obesity, increasing; triangle, overweight, increasing; circle, mid-normal weight, increasing; square, low-normal weight, increasing. (b) Male aged over 60. The trajectòries are as follows: diamond, obesity, stable; triangle, overweight, stable; circle, mid-normal weight, stable; square, low-normal weight, stable. (c) Female aged between 40 and 60. The trajectòries are as follows: diamond, obesity, increasing; triangle, overweight, increasing; circle, mid-normal weight, increasing; square, low-normal weight, increasing. (d) Female aged over 60. The trajectòries are as follows: diamond, obesity, increasing; triangle, overweight, stable; circle, mid-normal weight, stable; square, low-normal weight, stable.

In the trajectory analysis, each of the four groups was identified by four trajectory groups based on BIC (Bayesian Information Criterion scores). In Table 2, we presented the intercept, slope, and group membership for each trajectory group by age and sex. Fig 2a to 2d illustrate the trajectories of BMI in four distinct groups derived from group-based trajectory models; solid lines indicate the mean values of BMI. We differentiated the four trajectory groups as obesity, overweight, mid-normal weight, and low-normal weight based on the intercept displayed in Table 2. The linear slope was used to differentiate a stable or increasing trend. Mid-normal weight, regardless of a stable or an increasing trend, was the largest trajectory group for each age and sex group: 42.7%-47.2% (Table 2). Obesity was the smallest trajectory group, but was still composed of 4.4%-7.3% for the study population. The second largest trajectory group was the overweight group, except for women aged 40–60, in which low-normal weight was the second largest group.

**Table 2:** Estimates of growth curve parameters for body mass index trajectories.

Male aged over 60	Intercept, kg/m^2^		Linear Slope, kg/m^2^		Group membership, %
	Estimate	95% CI	Estimate	95% CI	
Obesity, stable	30.1	26.5, 33.7	0.001	-0.029, 0.031	7.3
Overweight, stable	25.9	23.6, 28.2	0.01	-0.004, 0.023	36.1
Mid-normal weight, stable	22.5	20.2, 24.7	-0.007	-0.02, 0.006	42.7
Low-normal weight, stable	18.8	16.2, 21.4	0.008	-0.016, 0.031	13.9
Male aged 40–60	Intercept, kg/m^2^		Linear Slope, kg/m^2^		Group membership, %
	Estimate	95% CI	Estimate	95% CI	
Obesity, increasing	31.1	27.1, 35.0	0.026	0.014, 0.038	6.1
Overweight, increasing	26.7	24.4, 29.0	0.035	0.03, 0.041	31.5
Mid-normal weight, increasing	23.5	21.2, 25.7	0.025	0.021, 0.029	46.6
Low-normal weight, increasing	19.9	17.4, 22.5	0.031	0.024, 0.038	15.7
Female aged over 60	Intercept, kg/m^2^		Linear Slope, kg/m^2^		Group membership, %
	Estimate	95% CI	Estimate	95% CI	
Obesity, increasing	32.7	28.4, 36.9	0.043	0.004, 0.082	5.8
Overweight, stable	27.6	24.9, 30.3	0.018	-0.002, 0.039	28.1
Mid-normal weight, stable	23.7	21.2, 26.3	0.0002	-0.015, 0.016	47.2
Low-normal weight, stable	19.8	16.9, 22.8	-0.017	-0.048, 0.006	18.9
Female aged 40–60	Intercept, kg/m^2^		Linear Slope, kg/m^2^		Group membership, %
	Estimate	95% CI	Estimate	95% CI	
Obesity, increasing	32.5	27.7, 37.3	0.079	0.064, 0.095	4.4
Overweight, increasing	27.3	24.6, 30.1	0.051	0.044, 0.058	21.9
Mid-normal weight, increasing	23.6	21.2, 25.9	0.032	0.027, 0.037	44
Low-normal weight, increasing	20.2	17.8, 22.7	0.012	0.007, 0.018	29.7

Abbreviations: CI, confidence interval.

Body mass index trajectories for the 4-group model over 19 years, adults from MJ cohort, 1998–2017. Solid lines indicate the mean values of body mass index (weight (kg)/height (m)^2^) for members in the groups; dashed lines, 95% confidence intervals. (a) Male aged between 40 and 60. The trajectories are as follows (from top to bottom): diamond, obesity, increasing; triangle, overweight, increasing; circle, mid-normal weight, increasing; square, low-normal weight, increasing. (b) Male aged over 60. The trajectories are as follows: diamond, obesity, stable; triangle, overweight, stable; circle, mid-normal weight, stable; square, low-normal weight, stable. (c) Female aged between 40 and 60. The trajectories are as follows: diamond, obesity, increasing; triangle, overweight, increasing; circle, mid-normal weight, increasing; square, low-normal weight, increasing. (d) Female aged over 60. The trajectories are as follows: diamond, obesity, increasing; triangle, overweight, stable; circle, mid-normal weight, stable; square, low-normal weight, stable.

HRs for overall mortality are displayed in Table 3 using the “mid-normal weight” trajectory group as the referent. Model 1 represents the model without any adjustment, whereas model 2 is fully adjusted for age, educational level, smoking status, alcohol consumption, and physical activity. Regardless of age and sex, the obesity group has the highest adjusted HR (men aged over 60: aHR = 1.26(CI = 1.12–1.42); men aged 40–60: aHR = 1.40(CI = 1.24–1.58); women aged over 60: aHR = 1.24(CI = 1.06–1.45); women aged 40–60: aHR = 1.33[CI = 1.14–1.57]). For men aged over 60, overweight is a protective factor in the unadjusted model (HR = 0.93, 95%CI = 0.87–0.99), but the effect disappeared after adjusting for lifestyle factors, age and education. Low-normal weight was significantly associated with mortality for all age and sex groups, except for women aged 40–60.

**Table 3:** Association between body mass index trajectories and all-cause mortality.

Male aged over 60	Death, number of death/ number of participants %	Model 1^a^		Model 2^b^	
		HR	95% CI	HR	95% CI
Obesity, stable	324/549, 59%	1.14*	1.01, 1.28	1.26***	1.12, 1.42
Overweight, stable	1544/2958, 52%	0.93*	0.87, 0.99	0.98	0.91, 1.04
Mid-normal weight, stable	1947/3582, 54%	1	referent	1	referent
low-normal weight, stable	683/1057, 65%	1.31***	1.20, 1.43	1.21***	1.11, 1.32
Male aged 40–60	Death, number of death/ number of participants %	Model 1^a^		Model 2^b^	
		HR	95% CI	HR	95% CI
Obesity, increasing	298/1983, 15%	1.46***	1.29, 1.65	1.40***	1.24, 1.58
Overweight, increasing	1255/10557, 12%	1.11**	1.03, 1.20	1.11**	1.04, 1.20
Mid-normal weight, increasing	1754/10357, 17%	1	referent	1	referent
low-normal weight, increasing	680/4943, 14%	1.30***	1.19, 1.42	1.21***	1.11, 1.32
Female aged over 60	Death, number of death/ number of participants %	Model 1^a^		Model 2^b^	
		HR	95% CI	HR	95% CI
Obesity, stable	180/417, 43%	1.16	0.99, 1.35	1.24**	1.06, 1.45
Overweight, stable	867/2089, 42%	1.10**	1.01, 1.20	1.02	0.93, 1.11
Mid-normal weight, stable	1455/3785, 38%	1	referent	1	referent
Low-normal weight, decreasing	598/1330, 45%	1.24***	1.13, 1.37	1.15**	1.04, 1.26
Female aged 40–60	Death, number of death/ number of participants %	Model 1^a^		Model 2^b^	
		HR	95% CI	HR	95% CI
Obesity, increasing	170/1710, 10%	1.53***	1.30, 1.80	1.33**	1.14, 1.57
Overweight, increasing	760/8641, 9%	1.32***	1.21, 1.45	1.16**	1.06, 1.27
Mid-normal weight, increasing	1232/18273, 7%	1	referent	1	referent
Low-normal weight, increasing	701/11711, 6%	0.88**	0.80, 0.96	1.05	0.96, 1.15

Abbreviations: CI, confidence interval; HR, Hazard ratio.

*p<0.05,

**p<0.01,

***p<0.0001.

^a^ Unadjusted model.

^b^ Fully adjusted model: adjusted for age, education level, smoking status, alcohol consumption and physical activity.

We conducted cause-specific analysis for competing risk analysis; the aHR are shown in Table 4 with a fully adjusted model. Likewise, the “mid-normal weight” trajectory was treated as the referent for each age and sex groups. Regardless of age and sex, respiratory disease had a significantly increasing mortality risk in low-normal weight groups (men aged over 60: aHR = 1.70(CI = 1.39–2.08); men aged 40–60: aHR = 1.85(CI = 1.31–2.61); women aged over 60: aHR = 1.94(CI = 1.45–2.58); women aged 40–60: aHR = 1.74(CI = 1.10–2.74)). In addition, a significantly increasing risk of mortality of cardiovascular disease in the obesity trajectory group was also found in all age and sex groups (men aged over 60: aHR = 1.34(CI = 1.06–1.69); men aged 40–60: aHR = 2.08(CI = 1.61–2.68); women aged over 60: aHR = 1.35(CI = 1.02–1.81); women aged 40–60: aHR = 1.62(CI = 1.09–2.41)).

**Table 4:** Association between body mass index trajectories and cause-specific mortality hazard ratio^a^.

Male aged over 60	low-normal weight, stable	Mid-normal weight, stable	Overweight, stable	Obesity, stable
Cancer	1.06 (0.90, 1.25)	1	1.01 (0.89, 1.13)	1.336*** (1.09, 1.64)
Cardiovascular disease	0.94 (0.77, 1.15)	1	0.994 (0.87, 1.14)	1.336*** (1.06, 1.69)
Respiratory disease	1.70*** (1.39, 2.08)	1	0.817** (0.68, 0.98)	1.027 (0.73, 1.46)
Others	1.33** (1.14, 1.55)	1	1.004 (0.89, 1.13)	1.232 (0.99, 1.53)
Male aged 40–60	low-normal weight, increasing	Mid-normal weight, increasing	Overweight, increasing	Obesity, increasing
Cancer	1.02 (0.92, 1.30)	1	1.061 (0.95, 1.18)	1.205 (0.99, 1.46)
Cardiovascular disease	1.12 (0.90, 1.40)	1	1.363** (1.16, 1.61)	2.079*** (1.61, 2.68)
Respiratory disease	1.85*** (1.31, 2.61)	1	1.002 (0.71, 1.41)	0.699 (0.32, 1.51)
Others	1.25** (1.07, 1.46)	1	1.07 (0.94, 1.22)	1.425** (1.15, 1.77)
Female aged over 60	low-normal weight, increasing	Mid-normal weight, increasing	Overweight, increasing	Obesity, increasing
Cancer	0.92 (0.75, 1.12)	1	1.01 (0.86, 1.19)	0.75*** (0.60, 0.93)
Cardiovascular disease	1.08 (0.89, 1.31)	1	1.10 (0.96, 1.22)	1.35* (1.02, 1.81)
Respiratory disease	1.94*** (1.45, 2.58)	1	1.19 (0.94, 1.21)	1.24 (0.70, 2.22)
Others	1.21* (1.04, 1.40)	1	1.10 (0.95, 1.26)	1.56** (1.24, 1.96)
Female aged 40–60	low-normal weight, increasing	Mid-normal weight, increasing	Overweight, increasing	Obesity, increasing
Cancer	0.96 (0.84, 1.09)	1	1.04 (0.91, 1.18)	1.02 (0.79, 1.32)
Cardiovascular disease	1.15 (0.90, 1.48)	1	1.50** (1.19, 1.88)	1.62* (1.09, 2.41)
Respiratory disease	1.74*** (1.10, 2.74)	1	1.11 (0.68, 1.82)	2.03 (0.98, 4.19)
Others	1.09 (0.92, 1.30)	1	1.23* (1.04, 1.45)	1.68** (1.29, 2.19)

^a^: Fully adjusted model: Adjusted for age, education level, smoking status, alcohol consumption and physical activity.

*p<0.05,

**p<0.01,

***p<0.0001.

The obesity trajectory group had contrary mortality risk of cancer disease comparing men and women aged over 60. For men, the “obesity, stable” group had higher cancer mortality risk (aHR = 1.34, 95% CI = 1.09, 1.64), but for women, the “obesity, decreasing” group had a decreased risk compared to the referent (aHR = 0.75, CI = 0.60, 0.93).

## Discussion

The present study identified BMI trajectories over 18 years in a large sample of adults older than 40 years of age and examined the association of various BMI trajectory groups with all-cause and cause-specific mortality risk. This is one of the largest studies with almost twenty years of follow-up to examine the grouping of BMI changes in adulthood objectively and repeatedly.

There are several key findings in our study. First, we identified four distinct BMI trajectory groups for all gender and age combinations: low-normal weight, mid-normal weight, overweight, and obesity. Second, CVD-related mortality was associated with overweight and obese trajectory in middle-aged men and women, but not in older age men and women. Third, obesity was a protective factor in cancer-related mortality in women but a risk factor in men aged over 60. Fourth, low-normal weight was a risk factor in respiratory-related mortality for all age and gender groups.

The distribution of populations in different trajectory groups and the change of pattern of trajectory groups over time in Taiwan are different from Western populations. Our study’s main advantage is the objective and repeated measurements of weight at various points in time. This allowed us to determine BMI trajectories throughout adulthood and to categorize BMI into distinct groups over time.

There are some major differences comparing the BMI trajectory in our sample and in Western populations. First, the majority of the population concentrates in the mid-normal weight group in Taiwan, whereas in Western populations, such as in the US, trajectories of overweight and obesity often account for 70%–80% of the sample [28–30]. Our data are similar to other Asian countries such as Japan [5], where the mid-normal weight group is composed of 67.1% of the population. Second, the body weight status does not seem to vary across the lifetime in our sample, which is similar to studies in Japan [5], but different in studies in the US and Austria [29–31]. Asian populations may be different from Western populations on the issue of BMI trajectory. Third, we showed an increasing pattern of groups aged between 40 and 60, which was not demonstrated in other studies because most of the studies that examined the BMI trajectory and mortality only used data from older adults, such as aged over 60 [28].

The result of all-cause mortality among the older population in our study is compatible with a study in Japan that showed the lowest mortality risk in normal and the overweight trajectory group [5]. Older populations tend to have a reverse J-shaped association of BMI with all-cause mortality, indicating that underweight and obesity are both important risk factor of mortality, as shown in US, Canada and Australia [32–34]. This so-called “obesity paradox,” that a higher BMI has protective association with all-cause mortality in older population [6,35], was also found in our sample of the Taiwanese population. Combining the finding of obesity as not being a protective factor for mid-aged adults in our sample, one possible mechanism may have been selection bias. Obese mid-aged adults might be more likely to die from CVD death if they have severe obesity-related CVD. This may result in a sample biased toward less risk of obesity-associated CVD death in older population. Our analysis also confirmed an association between CVD deaths and obesity in mid-aged adults.

In the present study, obesity was a protective factor in cancer-related mortality in women but not in men. Sex differences might occur due to the four following reasons:

(1) Estrogens could inhibit the growth of tumor, such as for esophageal cancer [36], liver cancer [37,38], and colon cancer [39,40]. Cancer cell progression rate is associated with sex hormones and with the altered hormone environment in an obese state [41]. After menopause, the level of estrogen decreases in women with normal weight; however, in overweight or obese woman, the adipose tissue can secret estrogen. The level of estrogen is significantly higher in postmenopausal obese women than in postmenopausal normal-weight women, and is also higher than in men [42], and may in turn protect obese older women from cancer.

(2) Sex-related differences at the genetic and molecular levels can affect the differences in the degree of response to chemotherapy [43,44]. Several commonly used chemotherapy agents, such as 5-fluorouracil, doxorubicin, cisplatin, and paclitaxel, all have a lower clearance rate in women, which leads to higher therapeutic efficacy but also to more severe toxicity [45], such as lung cancer [44,46], colon cancer [44], gastric and pancreatic cancers [44]. Sex differences in cancer-related mortality may result from better prognosis of cancer treatment in obese women.

In the present study, low-normal weight was a risk factor in respiratory-related mortality in all four groups. Several studies expressed similar results, such as in Spain, Finland, and New Zealand [47–49]. A meta-analysis found that, compared to normal BMI, underweight adults with COPD have an increased risk of mortality [50]. This is because lower BMI is associated with an accelerated lung function decline in adults, resulting in higher livelihood for COPD [50], and for other respiratory diseases as well, such as cystic fibrosis, asthma, and pneumonia [51,52]. A possible explanation is that reduced skeletal muscle mass—especially reduced diaphragmatic muscle mass—is associated with low pulmonary function because of the decreased strength of the respiratory muscles [53]. In a study of healthy adults in Korea [54], the parameter which represented pulmonary function—forced expiratory volume (FEV1)—was decreased in an underweight population compared to normal weight (Odds Ratio (OR) = 2.10, 95% CI = 1.98–2.21). Underweight adults who are at risk for respiratory diseases may consider pulmonary rehabilitation programs that aim to improve lung function in addition to increasing weight [54]. Another explanation is that genetic factors modulate both BMI and lung function. For example, several studies, such as in US, Norway and Iran, found a significant association between BMI and the fat mass and obesity-associated (FTO) genes in many general population studies [55,56]. Among the FTO genes, the rs8050136 minor allele was associated with both higher BMI and better lung function [57].

The present study has several limitations. First, the time between the measurements was 3 years, limiting information regarding changes in between. However, in our results, there is limited change of BMI over each three years; we do not think this limitation will affect our results regarding trajectory analysis. Second, although BMI is the most commonly used measure of adiposity, it has been criticized as not being able to distinguish between excess fat, muscle, water, or bone mass [58], nor does it provide any indication of the distribution of fat among individuals, such as visceral fat or cutaneous fat [58]. This may lead to misclassification bias. The term of normal weight obesity (NWO) has been used since 2008, whereas around 15% of people were classified as NWO [59]. However, this relatively small percentage population may not lead to severe bias since some studies, which pay attention to the representativeness of BMI to obesity, still announce that BMI is still a good index for cardio-metabolic risk [60]. However, it is still important for further research to focus on the representativeness of BMI to obesity.

(3) Our dataset did not measure some confounders that are associated with both BMI trajectory and certain causes of death, such as common genetic factors, such as FTO genes and comorbidities such as diabetes, hypertension, liver and kidney diseases that were not available in the analysis.

The effect of causal inference cannot be fully established. The literature on BMI trajectory and all-cause mortality, such as studies from Australia and Japan, have adjusted for confounders including diabetes and heart disease [5,30]. When comparing the results between unadjusted and adjusted, the result of all-cause specific mortality was generally similar [5,30]. Some of the roles of potential confounders such as diabetes might become mediators in a longitudinal study because people who are obese might also develop diabetes later on even if they do not have diabetes in the baseline. How these potential confounders might complicate the association between a longitudinal trajectory and mortality would require further analysis. Our study can offer an exploratory analysis only on the association, and warrants caution when interpreting our findings.

(4) Because the participation of MJ health screening is membership based, it is possible that this cohort may have had a slightly higher socioeconomic status than in the general Taiwanese population [22]. Our study population had a higher mean age in the group of 40–60 (55.2-year-old versus 47.2-year-old) compared to the whole Taiwanese population at year 2000 (the year of recruitment), but was similar in the group of 60+ (69.8-year-old versus 70-year-old). The findings of our study are not generalizable to the Taiwanese population.

In conclusion, our study highlighted the impact of the BMI trajectory on cause-specific mortality. In contrast to prior observations made regarding Western populations, BMI change over time remain unchanged in nearly all trajectory groups. In our research, both the low-normal weight and obese group had significantly higher mortality risk compared to the mid-normal weight group in adults aged 40 to 60 among both sexes; this indicates that no obesity paradox was found in this age group. In cause-specific mortality risk, low-normal weight is always related to high risk of respiratory disease-related death, whereas obesity seems to be a protective factor to cancer-related mortality risk in women aged over 60 but not in men. Our research suggests that, in mid-life, it still shows benefit to maintain a mid-normal weight in order to lower the mortality risk, whereas an intervention of a pulmonary rehabilitation program should be performed for low-normal weight groups. These findings could provide both clinical and public health approaches to body-weight management conducive to improving the survival of adults both in their mid- or late lives—particularly in Asian populations.
